# Professor Charpy

**Published:** 1911-08-19

**Authors:** 


### OBITUARY.

Professor Charpy, of Toulouse, an anatomist well known for his work on the morphology of the thorax and pelvis, died recently in that town from a chill following the ingestion of ice.

